# World Health Organization critical priority *Escherichia coli* clone ST648 in magnificent frigatebird (*Fregata magnificens*) of an uninhabited insular environment

**DOI:** 10.3389/fmicb.2022.940600

**Published:** 2022-08-11

**Authors:** Ana Carolina Ewbank, Danny Fuentes-Castillo, Carlos Sacristán, Fernanda Esposito, Bruna Fuga, Brenda Cardoso, Silvia Neri Godoy, Roberta Ramblas Zamana, Marco Aurélio Gattamorta, José Luiz Catão-Dias, Nilton Lincopan

**Affiliations:** ^1^Laboratory of Wildlife Comparative Pathology, Department of Pathology, School of Veterinary Medicine and Animal Sciences, University of São Paulo, São Paulo, Brazil; ^2^Departamento de Patología y Medicina Preventiva, Facultad de Ciencias Veterinarias, Universidad de Concepción, Chillán, Chile; ^3^One Health Brazilian Resistance Project (OneBR), São Paulo, Brazil; ^4^Centro de Investigación en Sanidad Animal (CISA-INIA), CSIC, Valdeolmos-Alalpardo, Spain; ^5^Department of Clinical Analysis, School of Pharmacy, University of São Paulo, São Paulo, Brazil; ^6^Department of Microbiology, Institute of Biomedical Sciences, University of São Paulo, São Paulo, Brazil; ^7^Refúgio de Vida Silvestre do Arquipélago de Alcatrazes – Instituto Chico Mendes de Conservação da Biodiversidade, São Paulo, Brazil

**Keywords:** pAmpC, ESBL, antimicrobial resistance, island, wildlife, One Health

## Abstract

Antimicrobial resistance is an ancient natural phenomenon increasingly pressured by anthropogenic activities. *Escherichia coli* has been used as markers of environmental contamination and human-related activity. Seabirds may be bioindicators of clinically relevant bacterial pathogens and their antimicrobial resistance genes, including extended-spectrum-beta-lactamase (ESBL) and/or plasmid-encoded AmpC (pAmpC), in anthropized and remote areas. We evaluated cloacal swabs of 20 wild magnificent frigatebirds (*Fregata magnificens*) of the Alcatrazes Archipelago, the biggest breeding colony of magnificent frigatebirds in the southern Atlantic and a natural protected area with no history of human occupation, located in the anthropized southeastern Brazilian coast. We characterized a highly virulent multidrug-resistant ST648 (O153:H9) pandemic clone, harboring *bla*_*CTX*–*M*–2_, *bla*_*CMY*–2_, *qnrB, tetB, sul1, sul2, aadA1, aac(3)-VIa* and *mdfA*, and virulence genes characteristic of avian pathogenic (APEC) (*hlyF, iroN, iss, iutA*, and *ompT*) and other extraintestinal *E. coli* (ExPEC) (*chuA, kpsMII*, and *papC*). To our knowledge, this is the first report of ST648 *E. coli* co-producing ESBL and pAmpC in wild birds inhabiting insular environments. We suggest this potentially zoonotic and pathogenic lineage was likely acquired through indirect anthropogenic contamination of the marine environment, ingestion of contaminated seafood, or by intra and/or interspecific contact. Our findings reinforce the role of wild birds as anthropization sentinels in insular environments and the importance of wildlife surveillance studies on pathogens of critical priority classified by the World Health Organization.

## Introduction

Antimicrobial resistance result from a naturally occurring ancient phenomenon that has been severely affected by anthropogenic activities such as use, misuse and overuse of antimicrobials in human and veterinary medicine, aquaculture and agriculture, and release of pharmaceutical manufacturing, domestic and agricultural waste into the environment (Wright, 2007; West et al., 2010; Yang et al., 2013; Michael et al., 2014). Worryingly, the issue of antimicrobial resistance leads to great healthcare, social and economical burdens worldwide, thus considered a quintessential One Health issue (Michael et al., 2014; Ewbank et al., 2021). *Escherichia coli* (order Enterobacterales) has been broadly suggested and used as a marker of environmental contamination and anthropogenic activity (Bonnedahl et al., 2009; Tenaillon et al., 2010). Extended-spectrum-ß-lactamase (ESBL)- and plasmid-encoded AmpC (pAmpC)-producing *E. coli* are classified as critical priority pathogens within the One health interface by the World Health Organization (WHO) (Tacconelli et al., 2018; Mughini-Gras et al., 2019).

Seabirds have been used as environmental bioindicators of ESBL/pAmpC-positive *E. coli* in remote locations due to their potential as sentinels of natural and anthropogenic-related changes to the marine ecosystem health (Hernandez et al., 2010; Hernández and González-Acuña, 2016; Ewbank et al., 2022). Given that clinically-relevant antimicrobial resistance genes are considered environmental pollutants and markers of environmental anthropization (Pruden et al., 2006; Jobbins and Alexander, 2015), most ESBL/pAmpC-producing *E. coli* studies have focused in synanthropic seabird species inhabiting anthropized environments (e.g., urban areas and dumpsites) (Bonnedahl et al., 2009; Atterby et al., 2016; Ahlstrom et al., 2018). Yet, insular biomes not inhabited by humans represent an informative setting in the study of the One Health chain of antimicrobial resistance by providing valuable insight into: (i) the occurrence, diversity, and dissemination of antimicrobial resistance genes (ARGs) and antimicrobial-resistant bacteria (ARB), such as ESBL/pAmpC-producing *E. coli*; (ii) the indirect anthropogenic effects over the environment (e.g., marine pollution); and (iii) the potential influence of biological and ecological characteristics of their local avian fauna (e.g., migration, use of coastal areas) (Hernandez et al., 2010; Ewbank et al., 2021, 2022).

Herein we analyzed cloacal swabs of 20 wild magnificent frigatebirds (*Fregata magnificens*; family Fregatidae) from an uninhabited archipelago located in southeastern Brazil, using microbiological techniques and whole genome sequencing (WGS) to investigate the occurrence, and phenotypic and genotypic characteristics of ESBL- and pAmpC-producing *E. coli* classified by WHO as critical priority pathogens (Tacconelli et al., 2018), and further identify and characterize their bacterial lineages, serotypes, resistome, plasmidome and virulome.

## Materials and methods

### Study area

**FIGURE 1:**
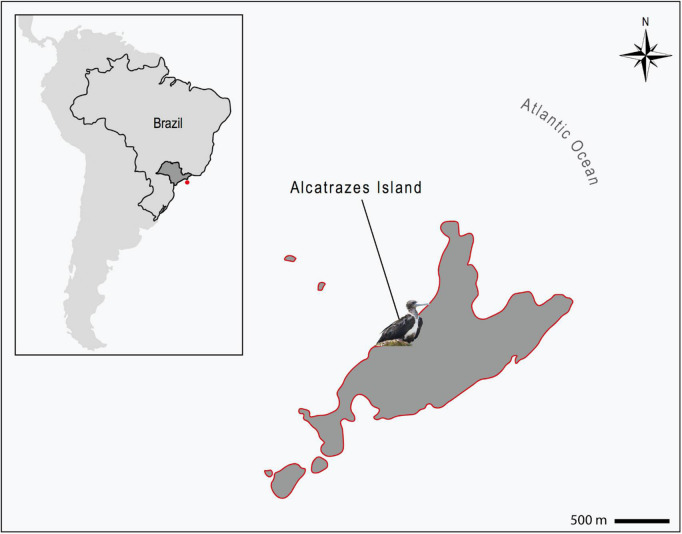
Location of the Alcatrazes Archipelago (red dot), territory of São Paulo state (dark gray), southeastern Brazil (inserted map). Magnificent frigatebird (*Fregata magnificens*) sampled in Alcatrazes island, part of the Alcatrazes Archipelago (lined in red). Scale: 500 m.

The Alcatrazes Island is the principal, among the five islands and four islets forming the Alcatrazes Archipelago (24° 05′ 44.69′′ S 45° 41′ 52.92′′ W), located at 36 km off the coast of São Sebastião, in São Paulo state, southeastern Brazil (Figure 1). The archipelago, including the Alcatrazes Island, have no records of onshore human occupation or tourist visitation, the latter limited to the offshore territory. In 1979, the Brazilian Navy started using the northeastern face of Alcatrazes Island as target for artillery practice. Later on, in 1987, the Tupinambás Ecological Station (Esec Tupinambás) was created, partially including the archipelago, and restricting visitation even more. In 2013, the Brazilian Navy moved its training grounds to a smaller island of Alcatrazes. Finally, in 2016, the archipelago and adjacent marine area (approximately 273 km^2^) were declared a conservation area - the Alcatrazes Archipelago Wildlife Refuge (Refuìgio de Vida Silvestre do Arquipeìlago de Alcatrazes - Refuìgio de Alcatrazes), focused specifically on the conservation of its local wildlife and flora, administered by the Chico Mendes Institute for Biodiversity Conservation (ICMBio), Brazilian Ministry of Environment (Instituto Chico Mendes de Conservação da Biodiversidade (ICMBio), 2017). This study was performed in full compliance with the Biodiversity Information and Authorization System (SISBIO 59150-4), Brazilian Ministry of Environment and the Ethical Committee in Animal Research of the School of Veterinary Medicine and Animal Sciences, University of São Paulo (Process number 1753110716).

### Sampling and bacterial identification

Twenty magnificent frigatebirds (17 adults and 3 juveniles) were sampled in the main island (Alcatrazes Island), in January 2020. The evaluated birds comprised nine males, eight females and three individuals of undetermined sex. All birds were captured with a butterfly net, manually restrained and immediately released after sample collection. The cloacal swabs were maintained in Amies transport medium containing charcoal and maintained at room temperature until processed (within 7 days). In order to select ESBL- and pAmpC-producing *E. coli* strains, cloacal samples were streaked onto ceftriaxone (CRO, 2 mg/L)-supplemented MacConkey agar plates) and incubated overnight at 35 ± 2°C. Bacterial isolates were identified by Matrix Assisted Laser Desorption Ionization Time of Flight Mass Spectrometry (MALDI-TOF MS, Bruker Daltonik, Germany).

### Antimicrobial susceptibility testing

Antimicrobial susceptibility was evaluated by the disc diffusion method using the following human and veterinary antimicrobials (Clinical and Laboratory Standards Institute [CLSI], 2018, 2019): amoxicillin/clavulanate, ceftriaxone, cefotaxime, ceftiofur, ceftazidime, cefepime, cefoxitin, imipenem, meropenem, ertapenem, enrofloxacin, ciprofloxacin, gentamicin, amikacin, chloramphenicol, trimethoprim-sulfamethoxazole, and tetracycline. The double-disc synergy test (DDST) was used for ESBL screening (EUCAST, 2017).

### Whole genome sequence analysis

The genomic DNA of the ESBL/pAmpC-positive *E. coli* strain was extracted using a PureLinkTM Quick Gel Extraction Kit (Life Technologies, Carlsbad, CA, United States) and a genomic paired-end library (75 × 2 bp), prepared using a Nextera XT DNA Library Preparation Kit (Illumina Inc., Cambridge, United Kingdom), according to the manufacturer’s instructions. The whole genome was sequenced on the NextSeq platform (Illumina). *De novo* genome assembly was performed with CLC Genomics Workbench 12.0.3. The draft genome sequence was automatically annotated using the NCBI Prokaryotic Genome Annotation Pipeline v.3.2. The MLST 2.0, PlasmidFinder 2.0, ResFinder 4.1, VirulenceFinder 2.0 and SerotypeFinder 2.0 databases available at the Centre for Genomic Epidemiology^1^ were used to identify, respectively, the multilocus sequence type (MLST), plasmid replicons, resistome, virulome and serotype. A prediction filter of ≥98 and 100% were set for sequence identity and coverage thresholds, respectively. Additionally, phylogroup analysis was performed using the ClermonTyping database^2^. The nucleotide sequence data reported is available in the DDBJ/EMBL/GenBank databases under accession number NZ_JAGYFD010000000. The *E*. *coli* AA18 strain genomic information is available on the OneBR platform under ID number ONE119^3^.

**FIGURE 2:**
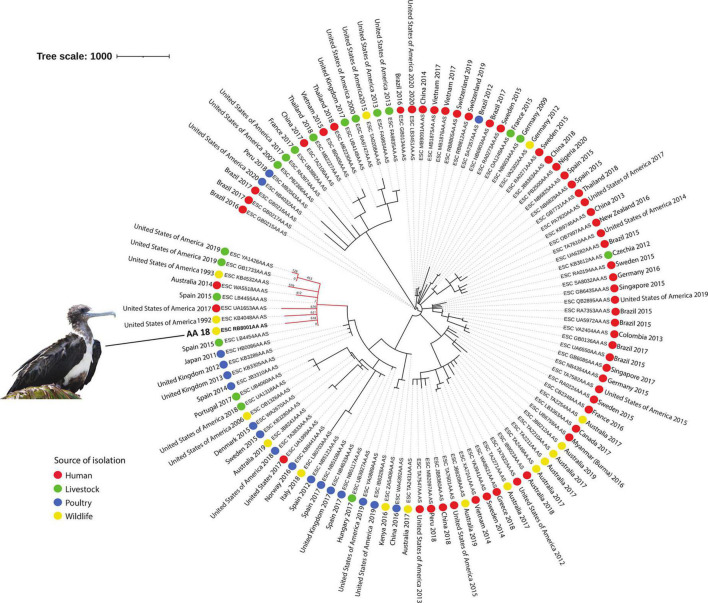
Phylogeny of CTX-M-2 and CMY-2-producing ST648 *E. coli* isolated from a magnificent frigatebird (*Fregata magnificens*) and global *E. coli* ST648. Each genome is shown in accordance to the source of isolation (colored circles), and country and year of isolation. In the red branch are the genomes that were phylogenetically closer to sample AA18. Tree scale: 1000.

The MSTree V2 tool from Enterobase^4^ was used to generate a minimum spanning tree based on the wgMLST scheme and 25,002 loci considering our *E. coli* isolate and an international collection of 107 *E. coli* strains belonging to ST648, selected according to source of isolation (colored circles), and country and year of isolation (Figure 2). iTOL v.6^5^ was used to edit and visualize the phylogenetic tree. An interactive version of the tree is available at https://itol.embl.de/tree/1791137681100671617229508.

## Results

Overall, we found an ESBL/pAmpC-producing *E. coli* prevalence of 5% (1/20) in the evaluated individuals. Phenotypically, the *E. coli* isolate (designated AA18 strain) presented a multidrug resistant (MDR) profile to amoxicillin/clavulanic acid, ceftiofur, cefoxitin, cefepime, aztreonam, trimethoprim-sulfamethoxazole, gentamicin, and tetracycline; remaining susceptible to carbapenems ertapenem, imipenem and meropenem (Clinical and Laboratory Standards Institute [CLSI], 2018, 2019). Regarding to genomic data, trimmed paired-end reads were assembled into 137 contigs, with 425,81 x coverage, and a G + C content of 49% (Andrews, 2010). Briefly, strain AA18 presented a genome size calculated as 5.4 million base pairs (bp), with 5,145 protein-coding sequences, 87 pseudogenes, 83 tRNAs, 3 rRNAs and 10 non-coding RNAs genomic analysis revealed that the isolate harbored genes *bla*_*CTX*–*M*–2_, *bla*_*CMY*–2_, *qnrB*, *tetB*, *sul1*, *sul2, aadA1*, *aac(3)-VIa* and *mdfA* in its resistome (Table 1).

**TABLE 1:** Genomic and epidemiological data of *E. coli* strain AA18.

Characteristics	*E. coli* strain AA18
Source	Cloacal swab
Genome size (Mbp)	5,4
No. of CDS^a^	5,145
G + C content (%)	57,25
tRNA (*n*)	83
rRNA (*n*)	3
Non-coding RNA (*n*)	10
Pseudogenes	87
CRISPR	2
MLST (ST)^b^	648
Serotype	O153:H9
Resistome	
β-lactams	*bla*_*CXT*–*M*–2_, *bla*_*CMY*–2_
Aminoglycosides	*aa(3)-VIa*, *aadA1*
Fluoroquinolones	*qnrB*
Sulphonamides	*sul1*, *sul2*
Tetracyclines	*tetB*
Macrolides	*mdfA*
Heavy metal	*bhsA, cusF, cutA, dsbAB, fetAB, fieF, glpF, mntPR, modE, nfsA, phnE, pitA, rcnR, robA, sitBCD, tehB, sodAB, ychH, yieF, yodD, zinT, znuA, zur*
Biocides	*cba, chuA, cma, cvaC, eilA, etsC, gad, hlyF, hra, ireA, iroN, iss, iucC, iutA, kpsE, kpsMII, lpfA, mchF, ompT, papC, sitA, terC, traT, tsh, yfcV*
Virulome	*acrE, cpxA, mdtEF, tehB, sugE, ydeOP*
Plasmidome	Col, IncFIB, IncFII
OneBR ID	ONE119
GenBank accession number	NZ_JAGYFD010000000

^a^CDSs, coding sequences. ^b^MLST, multilocus sequence type; ST, sequence type.

Multilocus sequence typing (MLST) and serotype analyses revealed that the isolate corresponded to ST648 and belonged to the O153:H9 group, respectively. Our isolate presented several relevant virulence genes characteristic of avian pathogenic (APEC) and other extraintestinal pathogenic *E. coli* (ExPEC), such as *chuA* (outer membrane hemin receptor), *kpsMII* (group 2 capsule synthesis), *fimC* (fimbriae type I), *sitA* (iron transport protein), and *traT* (transfer protein). Additionally, WGS analysis also identified genes encoding resistance to disinfectants (i.e., acridines, chlorhexidine, crystal violet, ethidium bromide, quaternary ammonium compounds, sodium dodecyl sulphate), heavy metals (i.e., lead, arsenic, copper, silver, antimony, zinc, tellurium, tungsten, cobalt, nickel, manganese, cadmium, mercury, iron, molybdenum, chromium, and vanadium), acid or basidic environment (i.e., H_2_O_2_, HCl and NaOH), and pesticide (glyphosate). The resistome, plasmidome and virulome are listed in Table 1.

Upon phylogenetic analysis, strain AA18 clustered with genomes from *E. coli* strains recovered from humans (Australia), livestock (Spain and United States), poultry (United States of America), and European herring gulls (*Larus argentatus*; United States of America), with a high variability ranging from 2 to 628 SNPs (Figure 2).

## Discussion

Herein, we found an overall prevalence of 5% (1/20) of ESBL/AmpC-positive *E. coli* isolates in magnificent frigatebirds of Alcatrazes Archipelago, southeastern Brazilian coast: a highly virulent MDR avian pathogenic *E. coli* (APEC) isolate of the pandemic high-risk ST648 clone (serotype O153:H9) harboring genes *bla*_*CTX*–*M*–2_ and *bla*_*CMY*–2_. To the authors’ knowledge, this is the first report of the ST648 clone and pAmp-EC in wild birds inhabiting insular environments.

The CTX-M-2 and CMY-2 enzymes are, respectively, the most prevalent CTX-M ESBL in South America and pAmpC beta-lactamase worldwide (Jacoby, 2009; Rocha et al., 2015), reported in a variety of epidemiological settings in Brazil (Rocha et al., 2015; Cunha et al., 2017; Melo et al., 2018; de Carvalho et al., 2020; Fernandes et al., 2020b). Among wild birds, *bla*_*CTX*–*M*–2_ and *bla*_*CMY*–2_ genes have been described in bacterial pathogens colonizing gulls, corvids and Eurasian magpie (*Pica pica*) in Europe (Loncaric et al., 2013; Stedt et al., 2015; Alcalá et al., 2016; Jamborova et al., 2017; Athanasakopoulou et al., 2021), and in gulls and bald eagles (*Haliaeetus leucocephalus*) from the Americas (Poirel et al., 2012; Báez et al., 2015; Atterby et al., 2016; Liakopoulos et al., 2016; Ahlstrom et al., 2018). In Brazil, *bla*_*CTX*–*M*–2_-positive bacteria have been detected in wild birds of prey and parrots, and in Magellanic penguin (*Spheniscus magellanicus*); whereas *bla*_*CMY*–2_-positive bacteria have been described in birds of prey (Sellera et al., 2017; Batalha de Jesus et al., 2019; de Carvalho et al., 2020).

The international clone ST648 is predominantly MDR and virulent, and one of the most commonly reported international sequence types (STs) in the human–animal–environmental interface worldwide, suggesting great host adaptation (Hu et al., 2013; Fernandes et al., 2018; de Carvalho et al., 2020). Of note, ST648 has been detected in wild birds from almost all continents, including Europe (Guenther et al., 2010; Schaufler et al., 2019), the Americas (Poirel et al., 2012; Báez et al., 2015), Asia (Hasan et al., 2012; Yang et al., 2016), and Oceania (Mukerji et al., 2019). In South America, this clone has been described in wild birds of prey in Brazil (Batalha de Jesus et al., 2019; de Carvalho et al., 2020) and gulls in Chile (Báez et al., 2015).

Strain AA18 carried several virulence genes of concern characteristic of highly pathogenic avian pathogenic *E. coli* (APEC) isolates: *cvaC* (colicin V), *fimC* (fimbriae type I), *hlyF* (hemolysin F), *iroN* (salmochelin), *iss* (increased serum survival), *iucC* (aerobactin production) *iut*A (ferric aerobactin receptor), *ompT* (outer membrane protein), *sitA* (iron transport protein), *tsh* (temperature-sensitive hemagglutinin) and *traT* (transfer protein) (Ewers et al., 2007; Sarowska et al., 2019). Additionally, we also found virulence genes characteristic of the other ExPEC: *chuA* (outer membrane hemin receptor), *kpsMII*, *papC* (outer membrane usher protein), and *yfcV* (major subunit of a putative chaperone-usher fimbria) (Grimwood et al., 2000; Kim, 2002; Sarowska et al., 2019). APEC strains may cause colibacillosis – multiple systemic and localized avian infection that may lead to high mortality and decreased production, capable of imposing severe economic losses to the poultry industry worldwide (Kemmett et al., 2014). Of note, some of the virulence factors found in our isolate were previously reported in ExPEC sampled from magnificent frigatebirds from the Alcatrazes Archipelago: *cvaC, fimH, hlyF, iroN, iss, iutA, ompT* and *papC* (Saviolli et al., 2016). Although APEC and other ExPEC strains are phylogenetically close, sharing some of the same virulence genes, APEC may carry other genes not common in other ExPEC isolates, such as those present in the colicin V (ColV) plasmid (Rodriguez-Siek et al., 2005; Bélanger et al., 2011). These characteristics suggest that APEC strains are potentially zoonotic, and could be a reservoir and source of virulence genes for other ExPEC strains (Ewers et al., 2007; Bélanger et al., 2011). In humans, APEC infections could take place through consumption of undercooked food from animal origin (especially retail poultry products), and direct contact with birds and their feces (Dziva and Stevens, 2008). Yet, despite the hypothetical zoonotic and pathogenic potential of our isolate (Ewers et al., 2014; Maluta et al., 2014; Sarowska et al., 2019), our findings must be carefully interpreted in light of the low prevalence of ESBL/AmpC-positive *E. coli* found herein (5%; 1/20) and the apparently healthy condition (with no signs of disease) presented by frigatebirds in Alcatrazes (also described by Saviolli et al., 2016). Furthermore, our strain also harbored genes encoding resistance to heavy metals, QACs and pesticides (Table 1), which may promote the development of AMR and co-selection of ARGs (Zou et al., 2014; Ramakrishnan et al., 2019; Mazhar et al., 2021).

Anthropization has been suggested as a driving factor in the epidemiology of ARGs in wildlife (Ahlstrom et al., 2018; Sacristaìn et al., 2020; Ewbank et al., 2021). Although occasionally visited or exploited for commercial guano harvesting until the mid-20*^th^* century, to this date, there are no reports of human occupation or settlements in the archipelago (Instituto Chico Mendes de Conservação da Biodiversidade (ICMBio), 2017). Nevertheless, Alcatrazes is located in the highly anthropized southeastern Brazilian coast, subjected to intense tourism activities, fishing, and oil exploitation, that also harbors the largest port complex (Santos Port), and oil and derivatives terminal in Latin America (Almirante Barroso Maritime Terminal - TEBAR). Of note, recent studies assessing antimicrobial resistance pollution in the marine ecosystem of the southeastern Brazilian coast showed that the local resistome is indeed under severe anthropogenic pressure (Fernandes et al., 2017, 2020a,b; Sellera et al., 2018a,b).

The Alcatrazes Archipelago is the largest insular bird breeding site of the southeastern Brazilian coast and the biggest breeding colony of magnificent frigatebirds in the southern Atlantic (Alcatrazes Island) (Instituto Chico Mendes de Conservação da Biodiversidade (ICMBio), 2017). Magnificent frigatebirds are non-synanthropic, non-migratory, and highly colonial seabird species that prefer insular over coastal environments, and also known for their particular feeding techniques (e.g., kleptoparasitism and fisheries interaction) (Saviolli et al., 2016; BirdLife International, 2021). Such characteristics infer that the studied Alcatrazes individuals most likely sustain very limited to no direct contact with humans, but that due to their philopatric (site fidelity) behavior, and limited roosting and nesting area of the island, are continuously interacting with the other frigatebird specimens and bird species using the area (especially with brown boobies (*Sula leucogaster*) and black vultures (*Coragyps atratus*), A.C. Ewbank, personal observation). Consequently, such close contact and active exchange of body fluids may be a possible route of infection by ESBL/pAmpC-positive *E. coli*, as seen in other avian pathogens (de Thoisy et al., 2009; Niemeyer et al., 2017).

Thus, in light of the above, we suggest that our isolate was likely acquired through one or more of the following: (i) indirect colonization by a bacterium released from human sources into the local marine environment (e.g., sewage) (Fernandes et al., 2017, 2018, 2020a,b); (ii) ingestion of contaminated seafood (Brahmi et al., 2015; Sellera et al., 2018a,b); and (iii) direct intra and/or interspecies contact (Ewbank et al., 2022).

Interestingly, according to the phylogenetic results, our isolate was not closely related to the selected ST648 isolates from other geographical regions or ecological sources included in the analysis. This indicates that, even though the origin of our isolate was likely related to human sources, this phylogenetic cluster seems to be restricted to the specific coastal/insular geographical area of Alcatrazes, southeastern Brazil. Nevertheless, additional studies in the region are necessary in order to confirm this hypothesis.

Previous studies have discussed the hypothetical potential of wild birds as reservoirs and disseminators of ARGs and ARB to insular biomes (Hernandez et al., 2010; Ewbank et al., 2021, 2022). Nevertheless, in spite of experimental studies assessing the shedding, contamination and potential transmission of ARGs and ARB by wild birds (Sandegren et al., 2018; Franklin et al., 2020), their potential role as dispersers under real-world conditions is still unknown. Our findings demonstrate that even in the absence of regular human presence, insular resistomes are indirectly pressured by anthropogenic activities, suggesting that contamination of the marine ecosystem and inter and/or intraspecific bird interactions should also be considered in the study of antimicrobial resistance in these biomes.

Herein we reported the genomic background of a critical priority *E. coli* strain belonging to the pandemic high-risk clone ST648 *E.coli* with a hypothetical zoonotic and avian pathogenic potential colonizing a wild magnificent frigatebird of an insular biome. Our findings reinforce, within a One Health perspective, the importance of surveillance studies of WHO critical priority pathogens in wildlife and the role of wild birds as anthropization sentinels in insular environments. Future studies evaluating the occurrence and diversity of ESBL/pAmpC-positive *E. coli* in magnificent frigatebirds on the Alcatrazes Archipelago should rely on continuous temporal sampling to assess a larger number of specimens, evaluate interacting species (i.e., brown boobies and black vultures), and environmental samples (i.e., sea water and soil), including local marine life (i.e., fish), in order to monitor these populations through a One Health approach and further elucidate the epidemiology of ESBL/pAmpC-positive *E. coli* in this insular environment.

## Data availability statement

The data presented in this study are deposited in the DDBJ/EMBL/GenBank repository, accession number NZ_JAGYFD010000000. The sequence has been released and is available at the repository: https://www.ncbi.nlm.nih.gov/nuccore/NZ_JAGYFD000000000.1.

## Ethics statement

This study was performed in full compliance with the Biodiversity Information and Authorization System (SISBIO 59150-4), Brazilian Ministry of Environment and the Ethical Committee in Animal Research of the School of Veterinary Medicine and Animal Sciences, University of São Paulo (Process no. 1753110716).

## Author contributions

AE: conceptualization, methodology, investigation, writing original draft, supervision, project administration, and funding acquisition. DF-C: methodology, investigation, writing original draft, and supervision. CS: conceptualization, methodology, investigation, funding acquisition, and writing original draft. FE: methodology, formal analysis, and writing original draft. BF and BC: methodology, and formal analysis. SG: conceptualization, investigation, resources, and funding acquisition. RZ and MG: investigation and resources. JC-D and NL: conceptualization, methodology, resources, writing–review and editing, supervision, project administration, and funding acquisition. All authors contributed to the article and approved the submitted version.

## Footnotes

1
http://genomicepidemiology.org/

2
http://clermontyping.iame-research.center/

3
http://onehealthbr.com

4
http://enterobase.warwick.ac.uk/species/index/ecoli

5
https://itol.embl.de
